# Ca^2+^ signals in plant immunity

**DOI:** 10.15252/embj.2022110741

**Published:** 2022-05-13

**Authors:** Philipp Köster, Thomas A DeFalco, Cyril Zipfel

**Affiliations:** ^1^ Institute of Plant and Microbial Biology and Zürich‐Basel Plant Science Center University of Zürich Zürich Switzerland; ^2^ The Sainsbury Laboratory University of East Anglia Norwich UK

**Keywords:** calcium, channel, ETI, immunity, PTI, Microbiology, Virology & Host Pathogen Interaction

## Abstract

Calcium ions function as a key second messenger ion in eukaryotes. Spatially and temporally defined cytoplasmic Ca^2+^ signals are shaped through the concerted activity of ion channels, exchangers, and pumps in response to diverse stimuli; these signals are then decoded through the activity of Ca^2+^‐binding sensor proteins. In plants, Ca^2+^ signaling is central to both pattern‐ and effector‐triggered immunity, with the generation of characteristic cytoplasmic Ca^2+^ elevations in response to potential pathogens being common to both. However, despite their importance, and a long history of scientific interest, the transport proteins that shape Ca^2+^ signals and their integration remain poorly characterized. Here, we discuss recent work that has both shed light on and deepened the mysteries of Ca^2+^ signaling in plant immunity.

## Graphical abstract

This review summarizes how calcium ions, and the proteins which transport them, are integrated into plant immune responses.

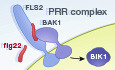

## The plant immune system

All eukaryotes use immune systems to protect themselves against potential pathogens. The plant immune system consists of two characterized perception layers: one that utilizes cell‐surface pattern recognition receptors (PRRs) to perceive extracellular immunogenic patterns, and another that relies on intracellular nucleotide‐binding leucine‐rich repeat (NLR) receptors that recognize pathogenic effectors inside the cell (Jones & Dangl, 2006).

**Figure 1:**
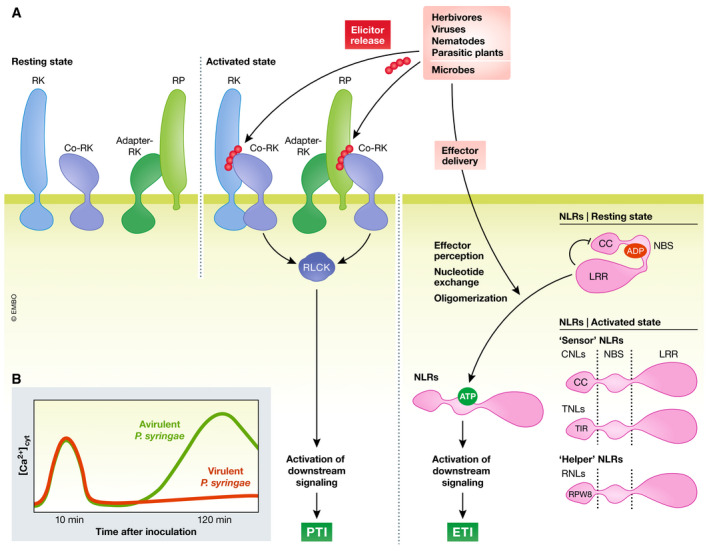
PTI and ETI induce cytoplasmic Ca^2+^ elevations RKs and RPs are PRRs residing at the PM. They form complexes with co‐receptors upon perception of molecular patterns originating from microbes, viruses, herbivores, parasitic plants, or damaged host cells. In turn, RLCKs are activated and released from the complexes to activate downstream signaling to induce pattern‐triggered immunity, of which Ca^2+^ release within few minutes after ligand perception is one facet. Microbes introduce effector proteins into host cells to disturb and overcome immune responses. Cytoplasmic NLRs sense the presence or activity of effectors to induce ETI. To this end, autoinhibition is released, ADP is changed to ATP and oligomerization of NLRs occurs, leading to downstream signaling and finally ETI (A). A significant cytoplasmic Ca^2+^ increase has been reported to occur in *Arabidopsis* leaves starting 1.5 h and peaking at about 2 h after infection with avirulent bacteria (B). Schematic Ca^2+^ signatures of *Arabidopsis* plants induced by bacterial infection as reported by Grant *et al* (2000) (B). RK: receptor kinase; co‐RK: coreceptor kinase; RP: receptor protein; RLCK: receptor like cytoplasmic kinase; NLR: nucleotide‐binding leucine‐rich repeat receptor; CC: coiled‐coil; TIR: toll/interleukin‐related; CNLs: CC‐NLRs; TNLs: TIR‐NLRs; RNLs: RPW8‐NRLs; NBS: nucleotide binding site; LRR: leucine‐rich repeats; PTI: pattern‐triggered immunity; ETI: effector‐triggered immunity, c[Ca2^+]^: cytoplasmic free Ca^2+^ concentration.

In the first layer of the plant immune system, apoplastic immunogenic elicitors such as pathogen‐, microbe‐, damage‐, or herbivore‐associated molecular patterns (PAMPs, MAMPs, DAMPs, or HAMPs, respectively) or immune‐modulating peptide phytocytokines are recognized by PRRs, which leads to defense responses termed pattern‐triggered immunity (PTI) (Boller & Felix, 2009; Yu *et al*, 2017; DeFalco & Zipfel, 2021). All plant PRRs described to date are receptor kinases (RKs) or receptor proteins (RPs) (Boutrot & Zipfel, 2017; Albert *et al*, 2020). RKs are characterized by a domain structure reminiscent of metazoan receptor tyrosine kinases (RTKs) (DeFalco & Zipfel, 2021); namely, a ligand‐binding extracellular domain (ECD), a single‐span transmembrane helix (TM) and a cytosolic protein kinase domain (Jamieson *et al*, 2018), while RPs lack a cytoplasmic kinase domain and instead form functional bipartite receptors with adapter RKs (Liebrand *et al*, 2013; Albert *et al*, 2015; Postma *et al*, 2016). Because of their domain architecture, plasma membrane (PM)‐localized PRRs (or their complexes) allow extracellular ligand binding to be communicated across the membrane into cytosolic signaling events. The molecular nature of elicitors varies, including proteins, lipids, and carbohydrates, and can be derived from either the potential pathogen or herbivore (*e.g*., MAMPs, PAMPs, or HAMPs) or the host plant, as in the case of macromolecules released upon cell damage (DAMPs) or secreted peptide phytocytokines (Gust *et al*, 2017). PRR ECDs are characterized by a variety of subdomains, including leucine‐rich repeat (LRR), epidermal growth factor‐like (EGF), lectin, and lysin motif (LysM) domains (Boutrot & Zipfel, 2017). The best‐studied PRRs to‐date are the LRR‐RKs FLAGELLIN‐SENSING 2 (FLS2) and EF‐TU RECEPTOR (EFR), which perceive the bacterial PAMPs flg22 and elf18, respectively (Gómez‐Gómez & Boller, 2000; Zipfel *et al*, 2006). Both FLS2 and EFR form stable ligand‐dependent complexes with common LRR‐RK co‐receptors of the SOMATIC EMBRYOGENESIS RECEPTOR KINASE (SERK) family, such as BRASSINOSTEROID‐INSENSITIVE 1‐ASSOCIATED KINASE 1 (BAK1, also called SERK3) (Chinchilla *et al*, 2007; Heese *et al*, 2007; Roux *et al*, 2011). Complex formation between PRRs and co‐receptors leads to phosphorylation events within the cytoplasmic kinase domains and the activation of receptor‐like cytoplasmic kinases (RLCKs), which directly phosphorylate and regulate target proteins in order to activate PTI (Liang & Zhou, 2018; DeFalco & Zipfel, 2021) (Fig 1A).

Pathogens introduce effectors into the host cytoplasm that promote pathogenicity, often by disturbing PTI (Jones & Dangl, 2006). To counteract this, plants rely on a second layer of immunity, in which intracellular NLR‐type receptors sense effectors and/or their activity, leading to effector‐triggered immunity (ETI). Interestingly, plant NLRs share a common architecture with those of animals, featuring a conserved nucleotide‐binding domain (NBD) and LRR domain, with variable accessory domains at both N and C termini (DeYoung & Innes, 2006; Jones *et al*, 2016; Baggs *et al*, 2017; van Wersch *et al*, 2020). NLRs are categorized based on their N‐terminal domains: coiled‐coil (CC)‐NLRs (CNLs), toll/interleukin‐related (TIR)‐NLRs (TNLs), or RPW8‐NLRs (RNLs). Of these NLRs, CNLs and TNLs function as sensors while RNLs function as helpers downstream of TNLs (Baggs *et al*, 2017; Wu *et al*, 2017; Jubic *et al*, 2019; Feehan *et al*, 2020). NLRs can be present in an inactive state, in which the LRR domain is likely autoinhibitory, and adenosine diphosphate (ADP) is bound to their NBD (Williams *et al*, 2011; Bernoux *et al*, 2016). Upon activation, ADP is exchanged to adenosine triphosphate (ATP) and autoinhibition is released (Fig 1A). In animals, NLR activation often leads to oligomerization via N‐terminal domains and the formation of large multimeric structures (Danot *et al*, 2009). A similar oligomerization mechanism has been long hypothesized for plant NLRs, but has only been recently corroborated by structural data that are discussed in detail below.

PTI and ETI have traditionally been viewed as independent pathways; however, at least some signaling components are shared by both layers of immunity (Thomma *et al*, 2011). Activation of either layer of the immune system triggers numerous overlapping cell signaling events, including Ca^2+^ fluxes, production of apoplastic reactive oxygen species (ROS), mitogen‐activated protein kinase (MAPK) cascades, transcriptional reprogramming, and phytohormone biosynthesis (Cui *et al*, 2015; Yu *et al*, 2017; Zhou & Zhang, 2020; DeFalco & Zipfel, 2021). ETI is generally also accompanied by a form of programmed cell death termed the hypersensitive response (HR) at the site of infection (DeYoung & Innes, 2006; Jones & Dangl, 2006), although HR‐like cell death is also induced by some forms of PTI (Wang *et al*, 2020). Recent work has further demonstrated that PTI and ETI are linked at transcriptional and/or molecular levels (Ngou *et al*, 2021; Pruitt *et al*, 2021; Tian *et al*, 2021; Yuan *et al*, 2021); however, the exact mechanisms governing linkage of these immune pathways remains to be elucidated fully. As changes in intracelluar Ca^2+^ levels have been well documented downstream of both PRR and NLR activation, Ca^2+^ signaling is thought to be key to both layers of the plant immune system (Seybold *et al*, 2014; Moeder *et al*, 2019).

## Ca^2+^ in immunity

Ca^2+^ is a universal second messenger in eukaryotes (Clapham, 2007). Owing to its cytotoxicity, cytosolic Ca^2+^ levels must be maintained at low (~10^−8^ to 10^−7^ M) levels in living cells, and thus Ca^2+^ is sequestered in intracellular stores (in plants, primarily the vacuole and the endoplasmatic reticulum, but also the vesicular compartments, the chloroplasts and mitochondria) or the apoplast via active transport, generating enormous electrochemical potential gradients across membranes (Clapham, 2007; Edel *et al*, 2017; Costa *et al*, 2018). Ca^2+^‐permeable channels can therefore generate rapid, transient increases in Ca^2+^ concentrations, which are in turn interpreted by a large suite of Ca^2+^‐binding sensor proteins that regulate diverse cellular processes (DeFalco *et al*, 2010). Ca^2+^ signaling is thus summarized in three steps: encoding (via stimulus‐triggered Ca^2+^ fluxes), decoding (via Ca^2+^ sensor proteins), and responses (via regulation of downstream cellular processes).

In plants, Ca^2+^ signaling is involved in all aspects of life, including growth regulation, development, abiotic stress responses, and reproduction (Kudla *et al*, 2018), as well as the establishment of beneficial plant‐microbe interactions (Tian *et al*, 2020). In this review, we focus on how cytoplasmic Ca^2+^ signals are encoded via transport across the PM during immune signaling.

Ca^2+^ influx and the oxidative burst (Doke, 1983, 1985; Apostol *et al*, 1989; Keppler *et al*, 1989) were among the first cellular responses to pathogen infection or elicitor treatment to be described (Atkinson *et al*, 1996; Levine *et al*, 1996; Zimmermann *et al*, 1997; Lecourieux *et al*, 2002). ROS production during the oxidative burst was eventually attributed to the activity of PM‐localized NADPH oxidases of the RESPIRATORY BURST OXIDASE HOMOLOGUE (RBOH) family (Torres *et al*, 2002); in the model plant *Arabidopsis thaliana* (hereafter, *Arabidopsis*), a single member, RBOHD, is responsible for ROS production in response to elicitors (Nühse *et al*, 2007; Zhang *et al*, 2007). In contrast, the molecular nature of the Ca^2+^ channel(s) involved in plant immunity remained comparably elusive for many years (Seybold *et al*, 2014).

Cytosolic Ca^2+^ signals evoked by treatment with various immunogenic elicitors were first measured in plant cell culture using Ca^2+^ radioisotopes, Ca^2+^‐sensitive dyes, or electrophysiological approaches (Atkinson *et al*, 1996; Levine *et al*, 1996; Gelli *et al*, 1997; Zimmermann *et al*, 1997). The development of genetically encoded Ca^2+^ indicators (GECIs) greatly expanded the possibilities for real‐time, kinetic analysis of Ca^2+^ fluxes in intact tissues upon infection or elicitor treatment. The first GECI deployed in plants was aequorin (AEQ) from *Aequoria victoria* (Knight *et al*, 1991), which forms a holo‐enzyme with its cofactor coelenterazine and emits light upon Ca^2+^‐binding. When challenged with either virulent or avirulent strains of the pathogenic bacterium *Pseudomonas syringae*, *Arabidopsis* plants expressing AEQ showed a first Ca^2+^ signal peak after ~10 min. A second, stronger, more persistent Ca^2+^ signal was seen after 1.5–2 h only with avirulent, ETI‐activating *P*. *syringae* (Grant *et al*, 2000; Kang *et al*, 2010; Hung *et al*, 2014). The similar kinetics of early Ca^2+^ elevation induced by *P*. *syringae* and that triggered by elicitors (Blume *et al*, 2000; Lecourieux *et al*, 2002) and the biphasic nature of the response to ETI‐inducing bacteria suggested that PTI and ETI may induce distinct Ca^2+^ signals (Fig 1B).

Subsequent analyses of AEQ‐expressing *Arabidopsis* plants have shown perception of diverse elicitors, including PAMPs, DAMPs, and phytocytokines, to be sufficient to elicit rapid Ca^2+^ signals (Ranf *et al*, 2008, 2011; Vadassery *et al*, 2009; Krol *et al*, 2010). Such PTI Ca^2+^ signaling requires functional PRRs and downstream signaling components, including RLCKs such as the RLCK‐VII/ AVRPPHB SUSCEPTIBLE 1 (PBS1)‐LIKE (PBL) family members BOTRYTIS‐INDUCED KINASE 1 (BIK1) and PBL1 (Li *et al*, 2014; Ranf *et al*, 2014; Monaghan *et al*, 2015). More recently, the deployment of fluorescent GECIs in plants has allowed for the analysis of elicitor‐induced Ca^2+^ signals at the cellular level. Such fluorescent GECIs include ratiometric (*e.g*., yellow cameleons) and intensiometric (*e.g*., GCaMPs and GECOs) sensors (Grenzi *et al*, 2021b; Waadt *et al*, 2021). Flourescent GECIs have been utilized to show that elicitor‐induced Ca^2+^ signals in leaves are oscillatory at the single‐cell level (Thor & Peiter, 2014; Keinath *et al*, 2015) and that in roots both elicitor application and laser ablation‐induced cell damage lead to the formation of Ca^2+^ transients (Keinath *et al*, 2015; Marhavý *et al*, 2019; Waadt *et al*, 2020).

## ROS and Ca^2+^—tightly linked second messengers

**Figure 2:**
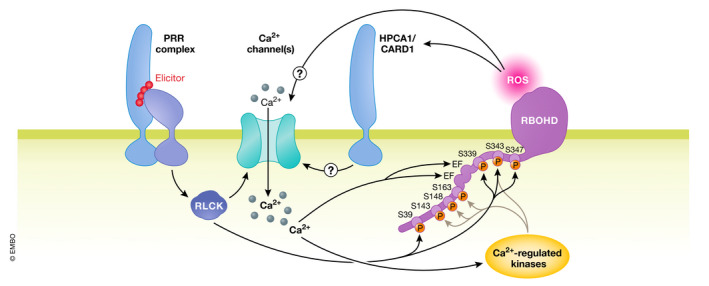
Ca^2+^ and ROS signals are tightly interconnected Upon activation of PRR complexes during PTI, RLCKs activate Ca^2+^ channels leading to cytoplasmic Ca^2+^ signals. Ca^2+^ ions can directly activate the NADPH‐oxidase RBOHD through binding to its N‐terminal EF‐hands, but also induce the activity of Ca^2+^‐regulated kinases that phosphorylate the cytoplasmic N terminus of RBOHD (indicated by grey arrows targeting RBOHD p‐sites). In addition, RLCKs directly phosphorylate the N terminus and thereby activate RBOHD (indicated by black arrows targeting RBOHD p‐sites). Reactive oxygen species derived from RBOHD activity can be perceived by cysteine pairs of the RK HPCA1/CARD1. This is required for H_2_O_2_ induced Ca^2+^ signals in *Arabidopsis*, the signaling pathway downstream of HPCA1 activation is not known.

There is extensive interplay between Ca^2+^ and ROS signaling (Gilroy *et al*, 2016); however, the initial PTI‐related Ca^2+^ signal triggered by *P*. *syringae* was shown to be only mildly reduced by treatment with the NADPH oxidase inhibitor DPI or catalase, while there was no effect on the longer‐term, effector‐triggered signal (Grant *et al*, 2000). Similarly, *rbohd* mutants showed a slight, quantitative defect in elicitor‐triggered Ca^2+^ signals when measured in seedlings (Ranf *et al*, 2011). In contrast, elicitor‐induced ROS production can be severely attenuated by treatment with Ca^2+^ channel blockers (Ranf *et al*, 2011). Elicitor perception can directly activate RBOHD via phosphorylation by BIK1 (Kadota *et al*, 2014; Li *et al*, 2014), suggesting a complex relationship between Ca^2+^ and ROS in immune signaling and a model wherein, upon elicitor perception, initial activation of RBOHD through PRR‐mediated phosphorylation primes the system for subsequent activation through Ca^2+^ signaling (Kadota *et al*, 2015) (Fig 2). Ca^2+^ not only activates RBOHD directly via its cytoplasmic Ca^2+^‐binding EF‐hand domains but also indirectly via Ca^2+^‐regulated kinase‐mediated RBOHD phosphorylation (Ogasawara *et al*, 2008; Dubiella *et al*, 2013). Interestingly, BIK1 and CALCIUM DEPENDENT PROTEIN KINASE 5 (CPK5) activate RBOHD through phosphorylation at distinct sites (Dubiella *et al*, 2013; Kadota *et al*, 2014; Li *et al*, 2014). While target residues have been described to be strictly required for PTI‐induced ROS bursts (Nühse *et al*, 2007), individual contribution from other phosphorylation sites and the impact of certain phosphorylation patterns remain to be uncovered.

A recent AEQ‐based screen for impaired H_2_O_2_‐induced Ca^2+^ signaling identified an LRR‐RK, HYDROGEN PEROXIDE INDUCED Ca^2+^ INCREASE 1 (HPCA1), as a putative ROS sensor (Wu *et al*, 2020a). Interestingly, HPCA1 was independently identified as CANNOT RESPOND TO DMBQ 1 (CARD1), which showed a loss of response to the quinone compound 2,6‐dimethoxy‐1,4‐benzoquinone (DMBQ), which regulates interactions with parasitic plants and also triggers HPCA1/CARD1‐dependent Ca^2+^ signaling (Laohavisit *et al*, 2020). Both the nature of the channel(s) that are regulated by HPCA1/CARD1, as well as the exact role of ROS in regulating Ca^2+^ signaling via such sensor(s) remain unclear. Interestingly, AEQ‐measured calcium signals in response to H_2_O_2_ were reduced in *cngc2* and *cngc4* mutants (Tian *et al*, 2019), suggesting that these channels may function downstream of ROS perception.

## Shaping immune signals via Ca^2+^ efflux

Ca^2+^ signals are generated via the coordinated action of channels and active transporters and involve influx from the apoplast and release from intracellular stores (Spalding & Harper, 2011; Edel *et al*, 2017; Resentini *et al*, 2021). In addition, plants possess three major families of proteins that mediate active Ca^2+^ transport out of the cytosol: Ca^2+^/H^+^ exchangers (CAXs), autoinhibited Ca^2+^‐ATPases (ACAs) and ER Ca^2+^‐ATPases (Geisler *et al*, 2000; Shigaki & Hirschi, 2000; García Bossi *et al*, 2020). ACA autoinhibition can be relieved by Ca^2+^/CaM‐binding, which allows for rapid feedback regulation of Ca^2+^ signals (Geisler *et al*, 2000). The PM‐localized ACA8 and its homolog ACA10 were identified as interactors of FLS2, and *aca8 aca10* mutants displayed quantitative defects in flg22‐induced calcium signals and compromised resistance to *P*. *syringae* infection (Frei dit Frey *et al*, 2012), as well as disturbed stomatal closure upon PAMP perception (Yang *et al*, 2017), suggesting that Ca^2+^ efflux across the PM to the apoplast shapes Ca^2+^ signaling during PTI.

**Figure 3:**
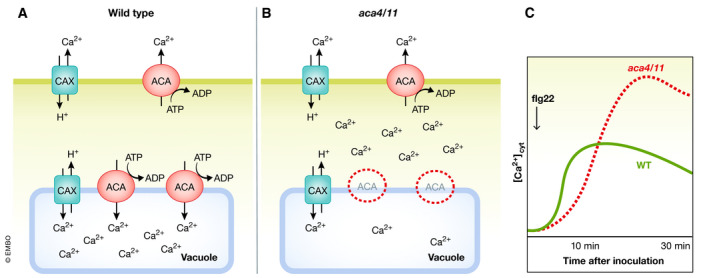
Disturbance of the Ca^2+^ efflux machinery impairs plant immunity Ca^2+^ exchangers (CAX) and autoinhibited Ca^2+‐^ATPase (ACAs) reside at the PM or tonoplast and establish low cytoplasmic Ca^2+^ concentrations and rapid termination of Ca^2+^ signals through export of the Ca^2+^ ions into the apoplast or vacuolar lumen (A). This function is disturbed in *Arabidopsis aca4 aca11* mutants, which consequently show an autoimmune phenotype (B). PTI induced Ca^2+^ signatures are compromised in those lines, with slower onset of the signal, and higher peak concentration and retarded reduction of the Ca^2+^ signals. Schematic Ca^2+^ signatures as reported by Hilleary *et al* (2020) (C).

Two tonoplast‐localized ACAs, ACA4 and ACA11, have also been implicated in immunity, as *aca4 aca11* mutants display autoimmune phenotypes and spontaneous cell death (Boursiac *et al*, 2010). Although *aca4 aca11* mutants have wildtype total calcium content (Boursiac *et al*, 2010), subsequent work has revealed that basal cytosolic calcium levels are elevated in *aca4 aca11* (Hilleary *et al*, 2020). Elicitor‐induced calcium signals also show elevated peaks in *aca4 aca11* mutants (Fig 3), which can be rescued by mis‐localization of PM ACAs to the tonoplast (Hilleary *et al*, 2020), indicating that transport of Ca^2+^ into the vacuole is critical to maintain Ca^2+^ homeostasis and modulate signaling during PTI.

## Plasma membrane‐localized Ca^2+^ channels involved in immunity

Extensive work has demonstrated that elicitor‐induced Ca^2+^ signals strictly require PM‐localized, Ca^2+^‐permeable channels, as treatment with blockers such as Gd^3+^ or La^3+^ abolishes such signals (Blume *et al*, 2000; Grant *et al*, 2000; Lecourieux *et al*, 2002; Kwaaitaal *et al*, 2011; Ranf *et al*, 2011; Maintz *et al*, 2014; DeFalco *et al*, 2017). While such studies clearly implicate Ca^2+^‐permeable channels as components of immune signaling, their nature has remained hidden. However, recent work has started to decipher how Ca^2+^ signals are generated upon immune activation, and the defense‐related roles of several classes of plant Ca^2+^ channels have begun to be characterized. Below, we discuss immunity‐related channel candidates by their phylogenetic groups rather than following a chronological order of identification or a strict PTI/ETI dichotomy.

### CNGCs—from strong phenotypes to complex regulation

One of the first families of potential Ca^2+^ channels identified in plants were the tetrameric cyclic nucleotide‐gated channels (CNGCs) (Köhler & Neuhaus, 1998). Plant CNGCs comprise large gene families (*e.g*., 20 members in *Arabidopsis*) (Mäser *et al*, 2001) and are named for their topology and domain organization, which are reminiscent of mammalian cyclic nucleotide‐gated (CNG) and hyperpolarization‐activated cyclic nucleotide‐modulated (HCN) families (Kaupp & Seifert, 2002; Matulef & Zagotta, 2003). Individual CNGCs have six transmembrane helices and cytosolic N and C termini, with the cyclic nucleotide‐binding domain (CNBD) located within the CNGC C terminus (Kaplan *et al*, 2007). While previous reports have indicated that the CNBDs of plant CNGCs may bind cyclic nucleotides (Baxter *et al*, 2008), and some electrophysiological analyses have indicated that application of cAMP or cGMP can promote CNGC activity (Leng *et al*, 2002; Zhang *et al*, 2007; Gao *et al*, 2014, 2016; Meena *et al*, 2019), it remains unclear whether cyclic nucleotides are *bona fide* agonists for plant CNGCs *in planta*. Furthermore, the existence of guanylate and adenylate cyclases (GCs and ACs) in plant proteomes is still under debate and will not be discussed in detail here. Indeed, while studies suggest multiple plant proteins, including RKs, to display GC activity (Qi *et al*, 2010; Turek & Irving, 2021), the low determined *in vitro* activities of the putative GCs and the position of their putative active sites within the kinase domains of RKs argues against a physiological relevance for such potential GC activity (Ashton, 2011; Bojar *et al*, 2014).

Nevertheless, extensive electrophysiological work over the past two decades has shown that at least some CNGCs form Ca^2+^‐permeable, non‐selective cation channels (Jarratt‐Barnham *et al*, 2021). CNGCs are directly regulated by the conserved Ca^2+^ sensor calmodulin (CaM), with one or more CaM‐binding domains (CaMBDs) found within the cytosolic C termini of all CNGCs examined to date (Arazi *et al*, 1999; Köhler & Neuhaus, 2000; Hua *et al*, 2003; Fischer *et al*, 2013, 2017; DeFalco *et al*, 2016a) as well as the N terminus of some CNGC isoforms (DeFalco *et al*, 2016a). Ca^2+^/CaM regulation of CNGCs is complex (DeFalco *et al*, 2016b) as a Ca^2+^‐independent IQ motif CaMBD at the C‐terminal end of the channel is essential for CNGC function (DeFalco *et al*, 2016a; Pan *et al*, 2019), with additional Ca^2+^‐dependent CaMBDs providing negative (feedback) regulation (DeFalco *et al*, 2016a; Pan *et al*, 2019; Tian *et al*, 2019).

Plant CNGCs are divided into four subfamilies based on phylogeny, with group IV CNGCs further divided into groups IVa and IVb (Mäser *et al*, 2001). The best‐studied CNGCs to‐date are the two *Arabidopsis* group IVb members, CNGC2 and CNGC4, which were first isolated as the *defense*, *no death* (*dnd*) or *HR‐like lesion mimic* (*hlm*) mutants *dnd1* and *dnd2/hlm1* (null mutants of *CNGC2* and *CNGC4*, respectively) (Clough *et al*, 2000; Balagué *et al*, 2003; Jurkowski *et al*, 2004). The *dnd* mutants were initially described to be defective in the induction of HR, despite still being able to carry out ETI to avirulent pathogens (Yu *et al*, 1998). These *dnd* mutants display numerous phenotypic defects, including dwarf morphology, delayed flowering, elevated concentrations of the phytohormone salicylic acid (SA), spontaneous cell death, and dis‐regulated auxin signaling (Clough *et al*, 2000; Balagué *et al*, 2003; Chan *et al*, 2003; Jurkowski *et al*, 2004; Chin *et al*, 2013; Chakraborty *et al*, 2021). In keeping with the immune‐related phenotypes of *dnd1/cngc2* mutants, CNGC2 was also suggested to be a mediator of Ca^2+^ fluxes in plant immunity, as production of the signaling molecule nitric oxide (NO) was reported to be reduced in *cngc2* mutants compared to WT plants after treatment with the PAMP lipopolysaccharide (LPS) (Ali *et al*, 2007). The same study used pharmacological inhibitors to implicate CaM, Ca^2+^ channels, and a NO synthase (NOS)‐type protein to be required for this process. Given the lack of mammalian‐type NOS enzymes in land plants (Santolini *et al*, 2017) and the myriad functions of CaM (DeFalco *et al*, 2010), results from such pharmacological studies must however be interpreted cautiously. Subsequent work using AEQ reporter lines suggested that CNGC2 is required for full Ca^2+^ signals in response to some but not all elicitors (Ma *et al*, 2012). Given the convergence of signaling downstream of diverse PRRs (Couto & Zipfel, 2016; Bjornson *et al*, 2021), it remains unclear how such specificity may be achieved. Interestingly, virus‐induced gene silencing (VIGS) of IVb isoforms in tomato compromised ROS production in response to flg22, further suggesting that these CNGCs may positively regulate PTI (Saand *et al*, 2015).

**Figure 4:**
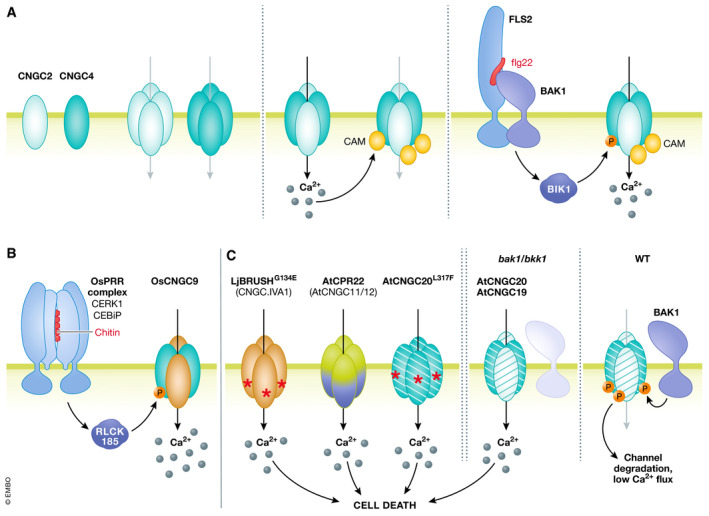
CNGCs fulfil diverse roles in plant immune signaling CNGCs form homo‐ or heterotertramers at the PM. *Arabidopsis* CNGC2 and CNGC4 homotetramers are inactive, but heterotetramers allow cation fluxes into the cytosol. Ca^2+^‐bound Calmodulin (CAM) inhibits those channels, generating a negative feedback loop. Upon initiation of PTI, activated BIK1 phosphorylates CNGC4 to release CAM‐mediated inhibition and to induce Ca^2+^ influx (A). In rice, PRR complexes activate RLCK185 upon ligand perception, which phosphorylates and thereby activates OsCNGC9. If the OsCNGC9 containing tetramer is homomeric or heteromeric is not known (B). In *Arabidopsis*, CNGC activity can lead to the induction of cell death via to date not resolved signaling pathways. CNGC19 and CNGC20 form complexes at the PM, and are phosphorylated by BAK1, which initiates degradation of the channels. In *bak1/bkk1* coRK mutants, accumulation of CNGC19/CNGC20 channels leads to Ca^2+^ influx, ultimately causing cell death.

Recently, loss‐of‐function *cngc2* and *cngc4* mutants were each isolated in an AEQ‐based forward genetic screen for compromised Ca^2+^ signaling upon flg22 treatment (Tian *et al*, 2019). Both mutants displayed defects in Ca^2+^ influx and ROS production after treatment with flg22 and exhibited compromised resistance to *P*. *syringae*. Remarkably, these phenotypes were however strictly dependent on high Ca^2+^ concentrations in the growth media, as *cngc2* and *cngc4* responses under low Ca^2+^ growth were indistinguishable from those of WT plants. Interestingly, PRR signaling mutants, such as *bik1*, do not display such conditional phenotypes (Li *et al*, 2014; Ranf *et al*, 2014; Monaghan *et al*, 2015). Detailed electrophysiological characterization of the heterologously expressed channels in *Xenopus laevis* oocytes found the single subunits to be inactive, while CNGC2‐CNGC4 heteromers produce strong currents (Tian *et al*, 2019), in keeping with a model wherein these channel subunits function together (Chin *et al*, 2013). CNGC2‐CNGC4 currents were inhibited by CaM; further experiments suggested that phosphorylation of the CNGC4 C terminus by BIK1 can partially release this negative regulation (Tian *et al*, 2019) (Fig 4A). This work further highlights the complex regulation to which CNGCs are likely subject, including by CaM, phosphorylation, and, potentially, ligand‐binding (Jarratt‐Barnham *et al*, 2021).

Both *cngc2* and *cngc4* mutants are hypersensitive to Ca^2+^ concentration in growth media (Chan *et al*, 2003; Chin *et al*, 2013), and their pleiotropic *dnd* phenotypes have been suggested to be caused by the mutant’s inability to take up Ca^2+^ from the apoplast into the cells in the vicinity of vasculature (Wang *et al*, 2017). Over‐accumulation of apoplastic Ca^2+^ and the resulting perturbations of both tissue‐ and cellular Ca^2+^ homeostasis may thus (at least partially) cause *cngc2* (and *cngc4*) phenotypes, though this will require further study to resolve fully. Given that PTI is not affected in *cngc2* and *cngc4* mutants grown at low Ca^2+^ concentrations (Tian *et al*, 2019), at such growth conditions—under which no growth defects also occur—other, currently unknown Ca^2+^ channels must also contribute to PTI (Dietrich *et al*, 2020). Recent studies reported a member of CNGC subfamily II, AtCNGC6, to be involved in the generation of Ca^2+^ signals during immunity after perception of the DAMP eATP, supporting the possibility of diverse CNGC subunits playing specific roles in plant immune responses (Duong *et al*, 2022).

### CNGCs and cell death

A genetic screen in rice (*Oryza sativa*, Os) recently identified loss‐of‐function mutants of Os*CNGC9* (a group III CNGC and homolog of *Arabidopsis* CNGC18) that displayed compromised resistance to rice blast disease and lesion‐mimic phenotypes after flowering (Wang *et al*, 2019b). PAMP‐induced Ca^2+^ currents across the PM were found to be strongly diminished in Os*cngc9* mesophyll cells compared to WT controls and, using an elegant heterologous reconstitution assay in mammalian cell culture, the authors demonstrated activation of the channel by OsRLCK185, a rice member of the RLCK‐VII/PBL family that functions downstream of chitin perception (Wang *et al*, 2019b) (Fig 4B). The autoimmune phenotypes of Os*cngc9* mutants are reminiscent of *Arabidopsis cngc2* and *cngc4*, it will therefore be interesting to determine whether such autoimmune phenotypes are due to these channels being guarded by NLRs and/or through perturbed Ca^2+^ homeostasis.

In contrast to the loss‐of‐function mutants described above, several gain‐of‐function CNGC mutants have also been isolated from genetic screens. These include several instances of (semi‐) dominant gain‐of‐function mutations that trigger autoimmunity such as *cpr22* (caused by expression of an in‐frame CNGC11/12 chimera) (Yoshioka *et al*, 2006; Urquhart *et al*, 2007) and *cngc20‐4* (caused by a leucine to phenylanaline mutation within one of the transmembrane helices of CNGC20) (Zhao *et al*, 2021) of *Arabidopsis* and the *brush* mutant of *Lotus japonicus* (hereafter, Lotus), which is caused by an N‐terminal glycine to glutamic acid mutation in the Lotus homolog of *Arabidopsis* CNGC19 (Chiasson *et al*, 2017) (Fig 4C). While such gain‐of‐function mutants must be interpreted cautiously, detailed study of these mutants suggests that dis‐regulated CNGCs can induce Ca^2+^‐ and SA‐dependent immunity and HR‐like cell death (Yoshioka *et al*, 2006; Urquhart *et al*, 2007; DeFalco *et al*, 2016a; Zhao *et al*, 2021), suggesting possible roles in ETI signaling and immunity more generally (Moeder *et al*, 2019).

CNGC20 has also recently been identified as a positive regulator of a specific form of autoimmunity (Yu *et al*, 2019). Loss of the SERK family co‐receptors BAK1/SERK3 and BAK1‐LIKE 1 (BKK1/SERK4) triggers constitutive cell death and seedling lethality (He *et al*, 2007; Kemmerling *et al*, 2007; Schwessinger *et al*, 2011). VIGS‐based screen revealed that this cell death is dependent on CNGC20 and to a lesser extent its close IVa homolog CNGC19 (Yu *et al*, 2019). This study further proposed a mechanism wherein SERKs phosphorylate the C terminus of CNGC20 to destabilize the channel in the absence of immunogenic stimuli, thereby precluding detrimental Ca^2+^ influx and cell death (Yu *et al*, 2019), adding an interesting component to the regulation of Ca^2+^ fluxes in immunity (Fig 4C). Whether or not recruitment of BAK1 into PRR complexes after elicitor recognition permits CNGC20 phosphorylation and therefore induces CNGC20 activity should be addressed in future studies. Recent work suggests a more complex role for Ca^2+^ in BAK1‐related cell death, as NLRs that mediate *bak1 bkk1* autoimmunity have been identified, including the NLR CONSTITUTIVE SHADE‐AVOIDANCE 1 (CSA1) (preprint: Schulze *et al*, 2021) and helper NLRs of the ACTIVATED DISEASE RESISTANCE 1 (ADR1)‐like family (Wu *et al*, 2020b). Strikingly, these ADR1‐type helper NLRs have recently been proposed to themselves act as Ca^2+^‐permeable cation channels (see detailed discussion below).

The other CNGC‐IVa member in *Arabidopsis*, *CNGC19*, was identified in a screen for genes whose expression was upregulated by mechanical wounding, and *cngc19* mutants were found to be more susceptible to *Spodoptera littoralis* caterpillars, likely due to impaired jasmonate (JA) and alipathic glucosinolate production (Meena *et al*, 2019). Interestingly, Ca^2+^ signals in response to the DAMP AtPep1 were also reduced in *cngc19* mutants (Meena *et al*, 2019), though other work using *cngc19 cngc20* protoplasts expressing GCaMP3 suggested that group IVa CNGCs are not required for the Ca^2+^ signals in response to flg22 (Yu *et al*, 2019). CNGC19 was also implicated in responses to the root‐colonizing mutualist endophytic fungus *Piriformospora indica* (Jogawat *et al*, 2020). *cngc19* mutants display clear phenotypes with respect to the symbiosis‐induced gain in growth rate; however, only minor defects in cytoplasmic Ca^2+^ rises after treatment with cell wall extracts from *P*. *indica* have been reported. This indicates the involvement of additional channel(s) (Jogawat *et al*, 2020).

An intriguing aspect of plant immune signaling is the propagation of electrical and second messenger‐based signals through the plant body, despite the obvious lack of any neurons or nervous system tissues in plants. In addition to reduced AtPep1 responses, *cngc19* mutants also displayed reduced systemic Ca^2+^ signals after mechanical wounding in the vasculature (Meena *et al*, 2019). Such signaling has long been associated with glutamate receptor‐like (GLR) channels, which are discussed below.

### GLRs – the long road to plant immunity

GLRs form a family of PM‐localized, ligand‐gated Ca^2+^ channels. Plant GLRs are named for their homology to metazoan ionotropic glutamate receptors (iGluRs), which are ligand‐regulated, homo‐ or heterotetrameric cation channels functioning in animal nervous systems. In *Arabidopsis*, GLRs form a 20‐member family that is sub‐divided into three clades: GLR1s, GLR2s and GLR3s; (Lam *et al*, 1998); individual members of the family have been implicated in various physiological processes (Wudick *et al*, 2018). GLRs feature a large, extracellular N‐terminal domain, which perceives amino acid ligands, three transmembrane helices and a short, cytosolic C terminus (Alfieri *et al*, 2020).

GLRs were first implicated in the generation of Ca^2+^ signals upon elicitor perception by pharmacological experiments using iGluR inhibitors, which reduced PAMP‐induced Ca^2+^ signals (Kwaaitaal *et al*, 2011). *Arabidopsis glr3.3* mutants were subsequently found to have compromised immunity toward the oomycete pathogen *Hyaloperonospora arabidopsidis*. In the same study, however, Ca^2+^ measurements in AEQ‐expressing *glr3.3* lines did not show reduced signals after treatment with oligogalacturonides (OGs), products of hydrolyzed host cell walls that act as DAMPs during *H*. *arabidopsidis* colonization (Manzoor *et al*, 2013). Similarly, *glr3.3* was also found to be more susceptible to *P*. *syringae* (Li *et al*, 2013), although formation of Ca^2+^ signals was not analyzed in that study. Together, such findings suggested a role for GLR3.3 in plant immunity which is distinct from its role in mediating the formation of the early Ca^2+^ signal. Indeed, ground‐breaking work from the labs of Ted Farmer and Simon Gilroy instead unraveled the role of GLR3s in the propagation of long‐distance signals and the formation of systemic immune responses. Multiple clade 3 GLRs were initially found to be required for the generation of electrical signals necessary for the induction of defense responses in distal tissues of plants after mechanical wounding or larval feeding on local leaves (Mousavi *et al*, 2013). Using *Arabidopsis* plants stably expressing GCaMP3, another study found that GLR3.3 and GLR3.6 are required for the generation of Ca^2+^ signals that propagate through the vasculature upon wounding or feeding (Toyota *et al*, 2018), and, correspondingly, *glr3.3 glr3.6* plants were shown to be more susceptible to *S. littoralis* (Nguyen *et al*, 2018). Simultaneous measurements of the electrical signals and cytoplasmic Ca^2+^ concentrations revealed that membrane depolarization preceded the rise of Ca^2+^ levels, a temporal sequence that was also observed in mesophyll cells after perceptrion of flg22 (Nguyen *et al*, 2018; Li *et al*, 2021). Such results highlight the specific role of clade 3 GLRs in systemic signaling. Taken together, those studies support a model wherein interconnected electrical, Ca^2+^ and ROS signals, as well as activity of the tonoplast‐localized cation channel TWO‐PORE CHANNEL 1 (TPC1) are required for effective long‐distance signal propagation in plants (Steinhorst & Kudla, 2014; Evans *et al*, 2016; Choi *et al*, 2017; Farmer *et al*, 2020; Johns *et al*, 2021).

It remains unclear whether clade 3 (or other) GLRs are also direct or indirect targets of PRR‐activated signaling pathways. It has been proposed that OG perception involves RKs of the WALL‐ASSOCIATED KINASE (WAK) family of epidermal growth factor (EGF)‐motif containing RKs (Brutus *et al*, 2010; Kohorn & Kohorn, 2012), while local Ca^2+^ signals in response to aphid feeding are BAK1‐ as well as GLR3.3‐ and GLR3.6‐dependent (Vincent *et al*, 2017). This suggests that the PRR signaling machinery may regulate GLR activity. Extracellular glutamate, which can act as a DAMP upon cell disruption, is also capable of inducing Ca^2+^ signals that are abolished in *glr3.3 glr3.6* mutants (Toyota *et al*, 2018; Shao *et al*, 2020), suggesting apoplastic amino acid(s) may act as agonist ligands for GLRs. The direct binding of glutamate to GLRs was further resolved through structural analysis of GLR3 ectodomains (Alfieri *et al*, 2020; Gangwar *et al*, 2021; Green *et al*, 2021). The role of clade 3 GLRs in systemic signaling has been recently reviewed in detail (Grenzi *et al*, 2021a), while the details of how ligand‐gating and/or PRR signaling coordinate the activation of GLR3 channel activity remain to be fully resolved.

**Figure 5:**
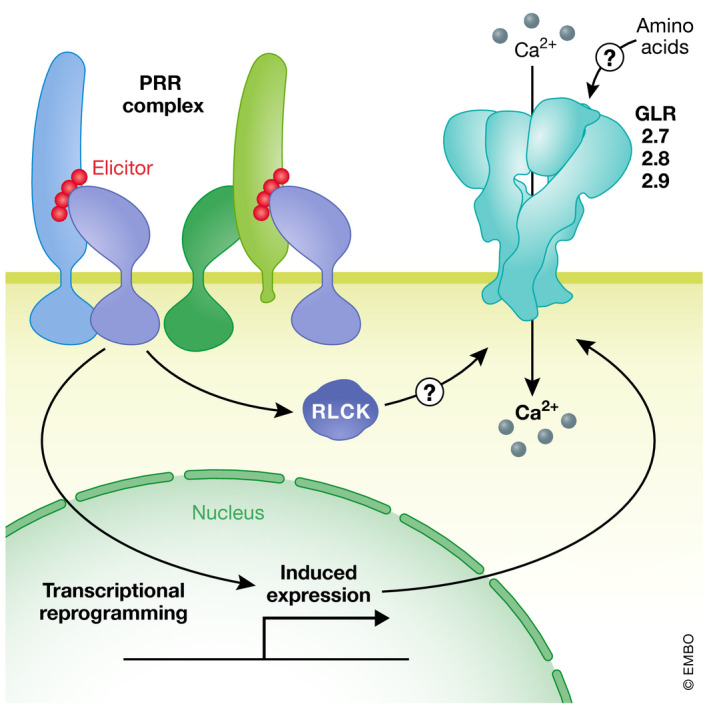
PRR signaling controls GLR2 abundance Upon perception of various elicitors, transcription of clade 2 GLRs is strongly induced. Activity of GLR2.7, GLR2.8, and GLR2.9 is required for complete PTI induced rapid Ca^2+^ influx, arguing for direct regulation of those channels in that process. The signaling pathway leading to this activation has not been resolved yet.

While clade 3 GLRs and their specific role in intercellular and long‐distance signaling are to date the best studied, other GLRs have also been recently found to play roles in the immune system. The *Arabidopsis* clade 2 GLRs GLR2.7 and GLR2.9 were recently identified in a large‐scale transcriptomic analyses as so‐called “core immunity response” (CIR) genes, which were transcriptionally upregulated in response to a panel of elicitors but not abiotic stresses (Bjornson *et al*, 2021) (Fig 5). GLR2.7 and GLR2.9 form a tandemly‐arranged, closely‐related cluster along with GLR2.8, and *glr2.7 glr2.8 glr2.9* triple mutants displayed defects in Ca^2+^ responses upon treatment with a variety of elicitors and reduced immunity against *P*. *syringae* (Bjornson *et al*, 2021). In keeping with their identification as CIR genes, these GLR2s were not found to contribute to Ca^2+^ signals during abiotic stress, suggesting that PTI involves common signaling components downstream of diverse elicitor/PRR complexes, but distinct from those involved in abiotic stress responses. As with GLR3s, how PRR complex activation mechanistically triggers rapid Ca^2+^ fluxes involving these GLR2s remains to be uncovered, as does the potential role for amino‐acid binding in this process.

### OSCAs, Ca^2+^ and stomatal gatekeeping

While the CNGC and GLR families of proteins were annotated shortly following release of the first sequenced plant genomes, the REDUCED HYPEROSMOLALITY, INDUCED CA^2+^ INCREASE (OSCA) family was only recently identified. OSCAs have nine transmembrane helices, with a short extracellular N terminus and a larger C terminus, and constitute a 15‐member family in *Arabidopsis* (Yuan *et al*, 2014). OSCA1.1 was identified in an AEQ‐based screen for regulators of Ca^2+^ signaling in response to osmotic stress (Yuan *et al*, 2014) and its homolog OSCA1.2 (also named CALCIUM PERMEABLE STRESS‐GATED CATION CHANNEL 1, CSC1) was identified through a heterologous screening of uncharacterized *Arabidopsis* transmembrane proteins for Ca^2+^ channel activity (Hou *et al*, 2014). Both OSCA1.1 and OSCA1.2/CSC1 were shown to be Ca^2+^‐permeable channels (Hou *et al*, 2014; Yuan *et al*, 2014), while subsequent structural, electrophysiological, and bioinformatic studies have revealed that OSCAs represent an evolutionarily conserved family of mechanosensitive, Ca^2+^‐permeable cation channels (Jojoa‐Cruz *et al*, 2018; Liu *et al*, 2018; Murthy *et al*, 2018).

**Figure 6:**
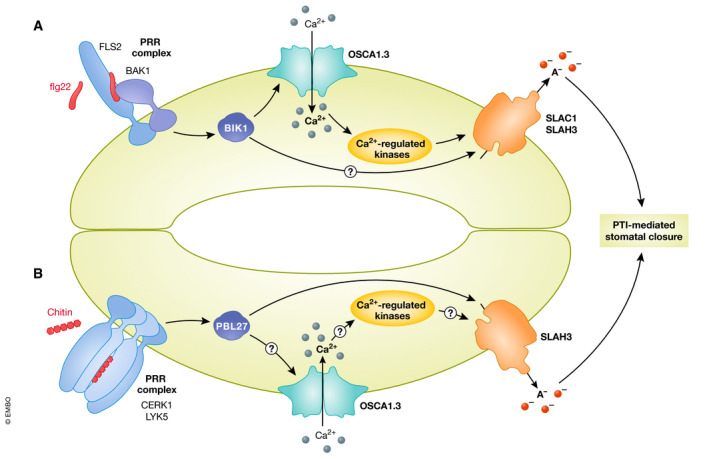
PRR signaling controls Ca^2+^‐dependent and Ca^2+^‐independent pathways leading to stomatal immunity Perception of bacterial flg22 leads to activation of BIK1 and phosphorylation of the Ca^2+^ channel OSCA1.3. Subsequent Ca^2+^ influx into the cytosol is required for guard cell closure. This closure is likely achieved through the activation of Ca^2+^‐regulated kinases, which in turn phosphorylate SLAC1 or SLAH3 anion channels. Upon channel activation of those channels, resulting ion fluxes cause turgor loss in the guard cell and stomatal closure (A). Perception of chitin by CERK1/LYK5 complexes activates the RLCK PBL27, which directly phosphorylates SLAH3, leading to stomatal closure (B).

In addition to systemic and long‐distance immune signaling, Ca^2+^ signaling also occurs at the single cell level in stomatal immunity. Stomata are gas‐exchange pores in the leaf epidermis that are formed by pairs of guard cells, with stomatal aperture controlled by changes in guard cell turgor (Lawson & Matthews, 2020). Aside from controlling gas exchange, stomata also serve as key points of entry for foliar pathogens (Melotto *et al*, 2017), and elicitor perception leads to rapid stomatal closure (Melotto *et al*, 2006; Desikan *et al*, 2008; Zeng & He, 2010). Stomatal closure is controlled by activation of SLOW ANION CHANNEL‐ASSOCIATED 1 (SLAC1) and/or SLAC1 HOMOLOGUE 3 (SLAH3), which mediate guard cell anion efflux, and which can be activated by Ca^2+^‐dependent or ‐independent phosphorylation cascades (reviewed in (Jezek & Blatt, 2017)). PTI signaling involves Ca^2+^ influx in guard cells (Thor & Peiter, 2014), and recently *Arabidopsis osca1.3 osca1.7* loss‐of‐function mutants were found to be defective in elicitor‐induced stomatal closure (Thor *et al*, 2020). The mechanism of the underlying core signaling pathway was duly unraveled, as OSCA1.3 was identified as a direct substrate of BIK1, which phosphorylates the channel on its N‐terminal cytosolic loop, providing a direct molecular connection from the activated PRR complex to the Ca^2+^ signal generation in guard cells (Fig 6A). PAMP treatment triggered phosphorylation of this BIK1‐dependent phosphosite (Benschop *et al*, 2007; Thor *et al*, 2020), and phosphorylation was found to promote the channel activity of OSCA1.3 in heterologous electrophysiological measurements (Thor *et al*, 2020). Ca^2+^ signaling defects in *osca1.3 osca1.7* mutants were also specific to guard cells, as signals in seedlings and epidermal tissues were unaffected. This study indicates a specific role of OSCA1.3 and OSCA1.7 in guard cells and stomatal immunity, and future studies may reveal whether other members of this family play additional roles in immunity, as well as how their potential mechano‐regulation contributes to such functions. Remarkably, another route of PRR signaling required for stomatal immunity was recently identified. Upon perception of chitin by CHITIN‐ELICITOR RECEPTOR KINASE 1 (CERK1)/ LYSM‐CONTAINING RECEPTOR‐LIKE KINASE 5 (LYK5) complexes, PBL27 directly phosphorylates the anion channel SLAH3 (Liu *et al*, 2019) (Fig 6B). Why different elicitors activate specific pathways to achieve the same physiological response, and whether the chitin induced pathway indeed functions without contribution of Ca^2+^ signaling, remains to be resolved.

In addition to the *osca* mutants defective in elicitor‐induced Ca^2+^ influx, disturbance of Ca^2+^ signals through loss of the ACA 8 and 10 cause loss of pathogen‐induced stomatal closure (Yang *et al*, 2017). Mutations in either of those Ca^2+^ pumps or their interactor BONZAI 1 (BON1) caused enhanced steady state Ca^2+^ signals and additionally failed to generate stimulus dependent stomatal Ca^2+^ oscillations due to retarded Ca^2+^ efflux after initial influx (Yang *et al*, 2017). Interestingly, the effect on guard cell Ca^2+^ fluxes in *osca1.3 osca1.7* mutants after flg22 application was quantitative (Thor *et al*, 2020), in contrast to the near‐complete loss of flg22‐induced stomatal closure in these mutants, while the defects in ACA8 and ACA10 activity still allowed the generation of Ca^2+^ signals but nevertheless prevented stomatal closure (Yang *et al*, 2017). Together, these studies suggest that minor perturbations in Ca^2+^ signals can trigger detrimental effects on downstream physiological processes.

### ANNs—atypical Ca^2+^ channels?

Annexins (ANNs) are small proteins occurring in both prokaryotes and eukaryotes and form a family of eight members in *Arabidopsis* (Laohavisit & Davies, 2011; Clark *et al*, 2012). Unlike other Ca^2+^‐permeable channels, ANNs are soluble proteins that lack transmembrane helices and instead reversibly bind negatively charged phospholipids, a process that is controlled by Ca^2+^ (Laohavisit & Davies, 2011). ANNs have previously been suggested either to regulate Ca^2+^ fluxes or provide Ca^2+^ transport activity themselves in response to H_2_O_2_ and salt stress (Laohavisit *et al*, 2012; Ma *et al*, 2019).

Recently, *Arabidopsis* ANN1 was identified as a positive regulator of local and systemic Ca^2+^ responses that are induced upon mechanical wounding and perception of *S*. *littoralis* oral secretions, with ANN1 loss‐of‐function or overexpression lines displaying enhanced or decreased susceptibility toward *S*. *littoralis*, respectively (Malabarba *et al*, 2021). Furthermore, *ann1* mutants were compromised in both transcriptional responses and JA production—phenotypes remarkably reminiscent of those reported for *cngc19* mutants (Meena *et al*, 2019). In this context, it will be an interesting target of future studies to parse how ANN1‐ and CNGC19‐mediated Ca^2+^ influx is able to distinguish between the induction of local and long‐distance signals.

In addition to those wound‐induced signals, ANN1 was also found to be involved in the generation of Ca^2+^ signals upon treatment of *Arabidopsis* with eATP (Mohammad‐Sidik *et al*, 2021), which is perceived as a DAMP by the L‐type lectin RK DOES NOT RESPOND TO NUCLEOTIDES 1/P2 RECEPTOR KINASE 1 (DORN1/P2K1) (Choi *et al*, 2014). The quantitative defect in eATP‐induced Ca^2+^ in *ann1* mutants suggests ANN1 as part of the signaling pathway downstream of PRR activation; however, it remains unclear whether ANN1 itself acts as a Ca^2+^ transporter, as well as how such activity is regulated. Furthermore, ANN1 was reported to interact with the chitin‐perceiving PRR CERK1 and thereby connects chitin perception and salt stress responses, a process in which ANN1 was previously characterized (Laohavisit *et al*, 2013; Espinoza *et al*, 2017). However, the underlying molecular mechanism remains to be resolved. Interestingly, ANN1 was independently identified as a mediator of *Arabidopsis* cold stress tolerance and was shown to positively regulate Ca^2+^ signals after cold shock (Liu *et al*, 2021). In this case, ANN1 Ca^2+^ transport activity was documented using electrophysiological characterization in *X*. *laevis* oocytes, with phosphorylation by the kinase OST1 having a positive effect on this activity (Liu *et al*, 2021). Whether similar regulatory phosphorylation of ANNs occurs in the context of immune signaling remains to be discovered, as does the mechanism by which activity of ANNs and GLRs are coordinated in the formation of long‐distance Ca^2+^ signals upon wounding.

### Ankyrin repeat domain proteins – a new class of Ca^2+^‐permeable channels in immunity?

Recently, LR14a, a wheat six‐transmembrane PM intrinsic protein with a N‐terminal cytoplasmic domain containing 12 ankyrin repeats was found to confer resistance to leaf rust in wheat (Kolodziej *et al*, 2021). Silencing of *LR14a* led to increased growth of the causal fungal pathogen *Puccinia triticina* and reduced induction of HR flecks. Interestingly, LR14a shares structural similarity with the mammalian protein TRANSIENT RECEPTOR POTENTIAL CHANNEL SUBFAMILY A MEMBER1 (TRPA1) (Suo *et al*, 2020; Kolodziej *et al*, 2021). TRPs are Ca^2+^‐permeable cation channel, suggesting a similar function of LR14a. LR14a was found to be required for the transcriptional induction of 160 genes upon infection with *P*. *tricitina* which were associated with the gene ontology term “Ca^2+^‐binding”. Overexpression of LR14a in *Nicotiana benthamiana* leaves induced a water‐soaking like phenotype indicative for osmotic disbalance, which could be prevented by the application of the Ca^2+^ channel blocker La^3+^ (Kolodziej *et al*, 2021). These findings support the possibility that LR14a acts as a Ca^2+^ channel, although electrophysiological characterization of the protein remains lacking.

Interestingly, another ankyrin repeat domain containing protein, *Arabidopsis* ACCELERATED CELL DEATH 6 (ACD6), is a positive regulator of cell death, as multiple *acd6* alleles were found to induce varying degrees of autoimmunity and have been subject of research for over 20 years (Rate *et al*, 1999; Lu *et al*, 2003). While the molecular basis of ACD6 action remained largely elusive, a recent study has documented ACD6‐induced ion channel activity upon heterologous expression in *X. laevis* oocytes (preprint: Zhu *et al*, 2021). Furthermore, autoimmunity of the *acd6‐1* allele could be abolished by growth at low [Ca^2+^], suggesting similar perturbances of Ca^2+^ homeostasis as reported for the *dnd* mutants (Chan *et al*, 2003; Chin *et al*, 2013; Wang *et al*, 2017; preprint: Zhu *et al*, 2021). ACD6 had been previously found to be associated with multiple RKs (Tateda *et al*, 2014; Zhang *et al*, 2017); however if and how this contributes to its regulation during immune responses has not been resolved.

### NLRs – wheels of death

PTI signaling immediately downstream of elicitor perception by PRRs involves a characteristic rapid and transient Ca^2+^ signal. Understanding elicitor‐triggered Ca^2+^ fluxes has been the focus of most studies of immunity‐related Ca^2+^ channels. ETI signaling, by contrast, involves long‐term, sustained Ca^2+^ signals (as discussed above). As outlined previously, PTI and ETI induce qualitatively similar signaling outputs, some of which (e.g., ROS, MAPK activation) have been shown to involve the same molecular components in both pathways (Kadota *et al*, 2019). It was thus reasonable to expect that similar channels were involved in Ca^2+^ signaling during both PTI and ETI, a supposition reinforced by the ETI‐like autoimmune and cell death phenotypes of several Ca^2+^ channel mutants, as discussed above. However, the landscape of Ca^2+^ channels in immunity was recently revealed to be more complex than previously thought.

**Figure 7:**
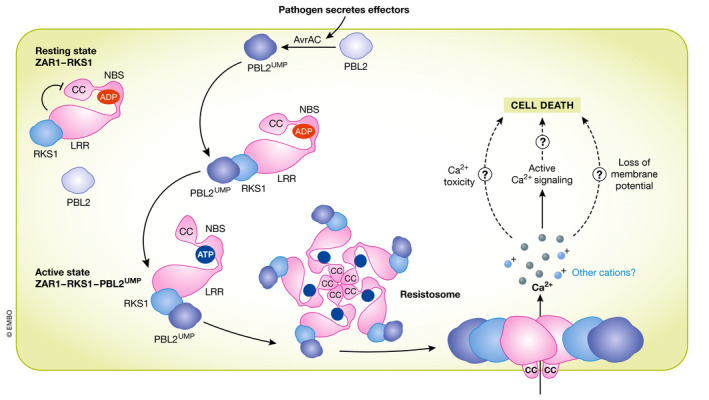
The ZAR1 resistosome forms a Ca^2+^‐permeable pore upon activation In the native state, ADP‐bound ZAR1 binds the RLCK RKS1. After delivery of the bacterial effector protein AvrAC, the RLCK PBL2 gets uridylated, which is in turn bound by the ZAR1‐RKS1 complex. Structural rearrangements lead to ADP exchange to ATP and relocalization of the CC domain. Pentamerization of ZAR1‐RKS1‐PBL2UMP complexes leads to the assembly of the resitosome multiprotein complex. The center of the complex is formed by the helices of the five CC domains and displays a funnel‐like form with a central pore. The funnel inserts into the PM and allows cation influx from the apoplast into the cytoplasm. This process is required for the initiation of the hypersensitive response. If cell death is achieved through Ca^2+^ toxicity, active Ca^2+^ signaling or a loss of membrane potential through the leak created by the resistosome, is not resolved yet. Besides ZAR1, also RNLs were found to form Ca^2+^ permeable pores after activation.

Plant NLRs have been long hypothesized to form large, multimeric complexes (as it the case in animals, (Jones *et al*, 2016)). This was finally shown to be the case with structural analysis of the complex of the CNL HOPZ‐ACTIVATED RESISTANCE 1 (ZAR1) and its RLCK interactors RESISTANCE‐RELATED KINASE 1 (RKS1) and PBL2 (Wang *et al*, 2019a, 2019c). Using cryo‐electron microscopy (cryo‐EM), the authors were able to resolve how activation of ZAR1 via uridylation of the decoy PBL2 by the bacterial effector AvrAC triggers subsequent exchange of ADP to dADP in the ZAR1 NBD, leading the complex to take a radially symmetrical, pentameric structure, termed a resistosome (Fig 7). Interestingly, the N‐terminal α‐helical domains of ZAR1 formed a funnel‐like domain within the resistosome, which was hypothesized to embed into membranes (Wang *et al*, 2019a). The overall resistosome structure resembled that of mammalian inflammasomes, and of the fungal toxin HET‐S, both of which create pores in membranes upon activation through terminal helical domains and thereby allow ion transport (highlighted in Dangl & Jones, 2019; Mermigka & Sarris, 2019), suggesting that this may be the case in plants. Subsequent work using a combination of detailed electrophysiological characterization and *in planta* Ca^2+^ measurements revealed the nature of the ZAR1 resistosome as a non‐selective cation‐channel with permeability to Ca^2+^ (Bi *et al*, 2021). This ion permeability is required for ZAR1‐induced cell death, which occurs through disintegration of the PM and cellular rupture (Bi *et al*, 2021).

In addition to the CNL ZAR1, TNLs have since been shown to also assemble into resistosome‐like structures (Ma *et al*, 2020; Martin *et al*, 2020), though no evidence yet indicates that these also form pores in membranes. Instead, helper NLRs of the RNL type such as ADR1 and NRG1.1, which function downstream of TNL sensors, were recently found to form oligomers and constitute ion pores through assembly of their α‐helical N‐terminal domains (Jacob *et al*, 2021). Auto‐activated forms of both NRG1.1 and ADR1 were found to function as Ca^2+^‐permeable channels *in planta* and to induce cell death upon controlled over‐expression. Similar to what had been reported for the ZAR1 resistosome (Bi *et al*, 2021), the cation permeability of NRG1.1 and ADR1 was dependent of the presence of negatively charged residues within the pore region of the protein complexes (Jacob *et al*, 2021).

The striking overall similarities found in the ZAR1 resistosome and the channels formed by NRG1.1 and ADR1 raise the question if the formation of ion‐permeable pores is indeed the general function of all helper NLRs. The physiological role of those channels will have to be analyzed in detail in future studies to resolve several open questions regarding channel‐like NLR functions in immunity. After strong, induced overexpression of (auto‐) activated helper NLRs, massive ion fluxes and rapid cell death have been documented (Jacob *et al*, 2021). This cell death is likely to be a consequence of the loss of ion homeostasis and resulting PM destabilization rather than Ca^2+^ signaling *per se*. It therefore remains to be seen whether, under natural infection conditions, effector‐triggered activation of NLR‐formed Ca^2+^ channels induces *bona fide* Ca^2+^ signals that are perceived by Ca^2+^ sensors to in turn induce physiological responses other than HR. Similarly, it will be critical to resolve how the channel‐like activities of NLRs are interwoven with those of classical Ca^2+^ channels, given both the ETI‐like phenotypes of numerous channel mutants and the interdependence of the BAK1‐related cell death on both ADR1‐type RNLs (Wu *et al*, 2020b) and CNGC20 (Yu *et al*, 2019).

Our understanding of NLR function continues to evolve rapidly, and recent parallel studies have reported NADase activity of the TIR domain of TNLs upon their activation (Horsefield *et al*, 2019; Wan *et al*, 2019; Ma *et al*, 2020). The mechanistic basis of this activity has been resolved with the structure of the TNL RPP1, wherein the tetrameric protein complex was found to form a holoenzyme (Ma *et al*, 2020). A similar tetramerization upon activation was also been recently reported for the *N. benthamiana* NLR RECOGNITION OF XopQ 1 (ROQ1) (Martin *et al*, 2020). How NADase activity regulates downstream signaling pathways remains to be fully characterized; however, it will be of great interest to determine if and how the resulting products (nicotinamide, adenosine diphosphate ribose (ADPR), and a variant of cyclic ADPR (v‐cADPR)) may modulate and/or induce Ca^2+^ fluxes. The same holds true for another recently‐reported enzymatic activity of TIR domain containing proteins: RESPONSE TO THE BACTERIAL TYPE III EFFECTOR PROTEIN HOPBA1 (RBA1) was recently found to produce 2′,3′‐cAMP/cGMP through hydrolysis of RNA and DNA molecules (preprint: Yu *et al*, 2021). Production of 2′,3′‐cAMP/cGMP appears to be required for TIR mediated signaling and cell death, but the exact function of those molecules will require further study.

Recently, plant genomes were found to encode proteins with similarities to necroptosis‐inducing MIXED LINEAGE KINASE‐DOMAIN LIKE (MLKL) proteins (Mahdi *et al*, 2020). In animals, those MLKL proteins are phosphorylated upon necroptosis to induce oligomerization. This causes them to translocate through membrane insertion of an N‐terminal four helix bundle called HeLo domain, which ultimately disturbs membrane integrity and causes cell death (Petrie *et al*, 2019). Interestingly, *Arabidopsis* MLKL3 and 4 were found to form tetramers, and loss of MLKL function led to severe defects in immunity toward the obligate biotrophic fungus *Golovinomyces orontii* via a TNL‐dependent pathway that does not involve the induction of cell death (Mahdi *et al*, 2020). Remarkably, chemical oligomerization of MLKL HeLo domains was found to be sufficient for the induction of cell death in *Arabidopsis* (Mahdi *et al*, 2020). How *Arabidopsis* MLKLs are regulated during immune responses, if their action also induces Ca^2+^ fluxes across the PM, and to what extent their functional mechanism is similar to that of the ZAR1 resistosome or the ADR1 type RNLs will be interesting topics for future studies.

## Conclusions and outlook: answers, yet more questions

The molecular basis of Ca^2+^ signaling during immune responses has been a major scientific question within plant biology for decades. As outlined in this review, numerous candidate channel proteins have been identified in recent years as contributing to PTI and/or ETI. However, despite this rapid increase in knowledge, critical questions remain unanswered, and the fact remains that the channel(s) responsible for the early Ca^2+^ transient during PTI is/are still largely unknown.

The study of immunity and Ca^2+^ signaling continues to benefit from tool development, and the modern, ever‐growing GECI repertoire has allowed for ever‐more detailed analyses of Ca^2+^ signals *in vivo*. However, we must remember that our conceptualization of Ca^2+^ signaling is at least partially defined by the GECIs we use, and may be too broad. It is possible that loss of individual Ca^2+^ channels evokes loss of individual Ca^2+^ signals within micro‐ and nanodomains, which are simply not resolved even by state‐of‐the‐art Ca^2+^ measurements.

It is remarkable that numerous channels from different families appear to contribute quantitatively to the rapid Ca^2+^ signal upon elicitor perception. These results beg the question of whether PRR‐mediated signaling cascades indeed target and regulate such a high number of individual channels. One possibility is that such a dividing and reunifying signaling architecture may allow for genetic robustness, although this remains to be explored. It is also possible that individual channels function in cell type‐specific manners, as has been at least suggested in the case of OSCAs in guard cells. It is also possible that each of the Ca^2+^ channels currently identified are indeed either quantitative contributors and/or regulators, while the channel(s) mediating the major influx still await identification. Indeed, the recent identification of channel families (*e.g*., OSCAs) or novel characterization of known proteins as potential channels (*e.g*., NLRs, ankyrin repeat domain proteins) indicates that there remains much to be discovered regarding Ca^2+^‐permeable channels in plants.

With regards to NLRs functioning as cation channels in ETI, future studies will have to find if they indeed generate Ca^2+^ signals that evoke specific downstream responses, or if their channel activity rather represents the loss of membrane impermeability, with Ca^2+^ influx just being a fellow traveler of cell death’s onset. In either case, it will be as well critical to resolve the role of “classical” Ca^2+^ channels in ETI signaling.

## Author contributions

**Philipp Köster:** Visualization; Writing—original draft; Writing—review & editing. **Thomas A DeFalco:** Visualization; Writing – original draft; Writing—review & editing. **Cyril Zipfel:** Funding acquisition; Writing—review & editing.

## Disclosure and competing interests statement

The authors declare that they have no conflict of interest.
